# On the Solution of NBVP for Multidimensional Hyperbolic Equations

**DOI:** 10.1155/2014/841602

**Published:** 2014-05-25

**Authors:** Allaberen Ashyralyev, Necmettin Aggez

**Affiliations:** Department of Mathematics, Fatih University, Buyukcekmece, 34500 Istanbul, Turkey

## Abstract

We are interested in studying multidimensional hyperbolic equations with nonlocal integral and Neumann or nonclassical conditions. For the approximate solution of this problem first and second order of accuracy difference schemes are presented. Stability estimates for the solution of these difference schemes are established. Some numerical examples illustrating applicability of these methods to hyperbolic problems are given.

## 1. Introduction

In the last decades, for the development of numerical methods and theory of solutions of the hyperbolic problems with nonlocal integral, Neumann and nonclassical conditions have been an important research topic in many natural phenomena. Solutions of this type of hyperbolic problems were investigated in [1–13]. These problems were studied in various directions: qualitative properties of solutions, spectral problems, various statements of boundary value problems, and numerical investigations.

For example, in [5] the nonlocal boundary value problem

(1) $$\begin{array}{c} \frac{d^{2}u\left(t\right)}{dt^{2}}+Au\left(t\right)=f\left(t\right),\;0\leq t\leq 1,\\ u\left(0\right)=\overset{n}{\underset{r=1}{\sum }}\alpha_{r}u\left(\lambda_{r}\right)+\varphi ,\;\;u_{t}\left(0\right)=\overset{n}{\underset{r=1}{\sum }}\beta_{r}u_{t}\left(\lambda_{r}\right)+\psi ,\\ 0<\lambda_{1}\leq \lambda_{2}\leq \cdots \leq \lambda_{n}\leq 1 \end{array}$$

was investigated. The stability estimates for the solution of the problem were established. The first order of accuracy difference schemes for the approximate solution of this problem was presented. The stability estimates for the solution of these difference schemes were established. Theoretical statements were supported by numerical examples.

The well-posedness of the Cauchy problem, Goursat problem, and boundary value problem for multidimensional hyperbolic equations have been studied extensively in a large cycle of papers (see, e.g., [14–21] and the references therein).

Actually, in paper [14], the Goursat problem for a linear multidimensional hyperbolic equation was investigated. Uniqueness of the solution and weak solvability of the Goursat problem were established.

In paper [15], the existence or nonexistence of global solutions of a multidimensional version of the first Darboux problem for wave equations with power nonlinearity in the conic domain was investigated.

In [16, 17], the solvability of an initial-boundary value problem for second order linear hyperbolic equations with a condition on the lateral boundary connecting the values of the solution or the conormal derivative of the solution with the values of some integral operator of the solution was studied. The existence and uniqueness theorems for regular solutions were proved.

In [18–20], the difference schemes for multidimensional hyperbolic equations were investigated. These methods were stable under the inequalities and contain the connection between the grid step sizes of time and space variables.

In [21], the authors develop a finite difference method (FDM) for a multidimensional coupled system of nonlinear parabolic and hyperbolic equations and prove the existence, stability, and uniqueness of its solution by a set of theorems. Finally, the proposed method was illustrated by a number of numerical experiments.

The study of difference schemes for hyperbolic equations with nonlocal conditions without using any necessary condition concerning the grid step sizes is of great interest. Such a difference scheme for solving the initial-value problem for abstract hyperbolic equations was studied for the first time in [22]. In the present paper, the following multidimensional hyperbolic equation

(2) ∂2u(t,x)∂t2−∑r=1m(ar(x)uxr)xr+σu(t,x)=f(t,x),x=(x1,…,xm)∈Ω, 0<t<1,

with nonlocal integral

(3) $$\begin{array}{c} u\left(0,x\right)=\overset{1}{\underset{0}{\int }}\alpha \left(\rho \right)u\left(\rho ,x\right)d\rho +\varphi \left(x\right),\;x\in \bar{\Omega },\\ u_{t}\left(0,x\right)=\overset{1}{\underset{0}{\int }}\beta \left(\rho \right)u_{t}\left(\rho ,x\right)d\rho +\psi \left(x\right),\;x\in \bar{\Omega }, \end{array}$$

and Neumann

(4) $$\begin{array}{c} {\frac{\partial u(t,x)}{\partial \vec{n}}|}_{S}=0 \end{array}$$

or nonclassical conditions

(5) $$\begin{array}{c} {u(t,x)|}_{S_{1}}=0,\\ {\frac{\partial u(t,x)}{\partial \vec{n}}|}_{S_{2}}=0,\;0\leq t\leq 1, \end{array}$$

under the assumption

(6) $$\begin{array}{c} \left|1+\overset{1}{\underset{0}{\int }}\alpha \left(s\right)\beta \left(s\right)ds\right|>\overset{1}{\underset{0}{\int }}\left(\left|\alpha \left(s\right)\right|+\left|\beta \left(s\right)\right|\right)ds \end{array}$$

is considered. Here, *Ω* is the unit open cube in the *m*-dimensional Euclidean space *R* ^*m*^ with boundary *S* = *S*_1_ ∪ *S*_2_, $\bar{\Omega }=\Omega \cup S$ ,  *a*_*r*_(*x*) (*x* ∈ *Ω*), *φ*(*x*), $\psi (x)\;\;(x\in \bar{\Omega })$ , and *f*(*t*, *x*)(*t* ∈ (0,1), *x* ∈ *Ω*) are given smooth functions, and *a*_*r*_(*x*) ≥ *a* > 0. $\vec{n}$ is the normal vector to *Ω*.

The first and second order of accuracy difference schemes for multidimensional hyperbolic problem (2) are presented. The schemes are shown to be absolutely stable. It is naturally seen that the second order difference schemes are much more advantageous than the first order ones.

## 2. Stability of First Order of Accuracy Difference Scheme

For approximately solving problem (2), first order of accuracy difference scheme

(7) τ−2(uk+1−2uk+uk−1)+Auk+1=fk, fk=f(tk+1),tk+1=(k+1)τ, 1≤k≤N−1, Nτ=1,u0=∑j=1Nα(tj)ujτ+φ,(I+τ2A)τ−1(u1−u0)=∑j=1Nβ(tj)(uj−uj−1)+ψ

is considered. A study of discretization of the nonlocal boundary value problem also permits one to include general difference schemes in applications, if differential operator in space variables *A* is replaced by difference operator *A*_*h*_ that acts in a Hilbert space and is uniformly self-adjoint positive definite in *h* for 0 < *h* ≤ *h*_0_.

The stability estimates of solution of difference scheme (7) are established under the assumption

(8) $$\begin{array}{c} 1>\overset{N}{\underset{j=1}{\sum }}\left|\alpha \left(t_{j}\right)\right|\tau \overset{N}{\underset{j=1}{\sum }}\left|\beta \left(t_{j}\right)\right|\tau +\overset{N}{\underset{j=1}{\sum }}\left|\alpha \left(t_{j}\right)\right|\tau +\overset{N}{\underset{j=1}{\sum }}\left|\beta \left(t_{j}\right)\right|\tau . \end{array}$$

**Lemma 1**

The following estimates hold [23]:

(9) $$\begin{array}{c} {||R||}_{H\mapsto H}\leq 1,\;\;{||\tilde{R}||}_{H\mapsto H}\leq 1,\\ {||R{\tilde{R}}^{-1}||}_{H\mapsto H}\leq 1,\;\;{||\tilde{R}R^{-1}||}_{H\mapsto H}\leq 1,\\ {||\tau A^{1/2}R||}_{H\mapsto H}\leq 1,\;\;{||\tau A^{1/2}\tilde{R}||}_{H\mapsto H}\leq 1, \end{array}$$

where

(10) $$\begin{array}{c} R={\left(I+i\tau A^{1/2}\right)}^{-1},\;\;\tilde{R}={\left(I-i\tau A^{1/2}\right)}^{-1}. \end{array}$$

**Lemma 2**

The operator

(11) T=I+∑j=1Nβ(tj)τ2R2R~2(Rj+R~j)−∑j=1Nα(tj)τ2[Rj−1−R~j−1]−∑j=1Nα(tj)τ∑j=1Nβ(tj)τ(RR~)j+2

has an inverse

(12) T−1={I+∑j=1Nβ(tj)τ2R2R~2(Rj+R~j) −∑j=1Nα(tj)τ2[Rj−1−R~j−1] −∑j=1Nα(tj)τ∑j=1Nβ(tj)τ(RR~)j+2}−1

and the following estimate is satisfied:

(13) ||T−1||H→H≤(1)×(1−∑j=1N|α(tj)|τ∑j=1N|β(tj)|τ       −∑j=1N|β(tj)|τ−∑j=1N|α(tj)|τ)−1.

**Proof**

Using formula (11) and the triangle inequality, we can write

(14) ||T||H→H ≥1−∑j=1N|α(tj)|τ∑j=1N|β(tj)|τ||(RR~)j+2||H→H  −∑j=1N|β(tj)|τ12||R2R~2||H→H(||Rj||H→H+||R~j||H→H)  −∑j=1N|α(tj)|τ12[||Rj−1||H→H+||R~j−1||H→H].

Applying the triangle inequality and estimates (9), we get

(15) ||T||H→H≥1−∑j=1N|α(tj)|τ∑j=1N|β(tj)|τ−∑j=1N|β(tj)|τ−∑j=1N|α(tj)|τ.

Thus, estimate (13) follows from this estimate.  Lemma 2 is proved. The following theorem on the stability estimates for the solution of difference scheme (7) is established.

**Theorem 3**

Let *φ* ∈ *D*(*A*), *ψ* ∈ *D*(*A* ^1/2^), and (8) hold. Then, for the solution of difference scheme (7) the following stability estimates hold:

(16) $$\begin{array}{c} \underset{0\leq k\leq N}{\max}{||u_{k}||}_{H}\leq M\left\{\overset{N-1}{\underset{s=1}{\sum }}{||A^{-1/2}f_{s}||}_{H}\tau +{||A^{-1/2}\psi ||}_{H}+{||\varphi ||}_{H}\right\}, \end{array}$$

(17) max⁡1≤k≤N||τ−1(uk−uk−1)||H+max⁡0≤k≤N||A1/2uk||H ≤M{∑s=1N−1||fs||Hτ+||ψ||H+||A1/2φ||H},

(18) max⁡1≤k≤N−1||τ−2(uk+1−2uk+uk−1)||H+max⁡0≤k≤N||Auk||H ≤M{∑s=2N−1||fs−fs−1||H+||f1||H+||A1/2ψ||H+||Aφ||H},

where *M* is independent of *f*_*s*_, 1 ≤ *s* ≤ *N* − 1, and *φ*,  *ψ*.

**Proof**

First, we obtain formula for the solution of difference scheme (7). For the solution of difference scheme

(19) $$\begin{array}{c} \tau^{-2}\left(u_{k+1}-2u_{k}+u_{k-1}\right)+Au_{k+1}=f_{k},\\ f_{k}=f\left(t_{k+1}\right),\;t_{k+1}=\left(k+1\right)\tau ,\\ 1\leq k\leq N-1,\;N\tau =1,\\ u_{0}=\mu ,\;\;\left(I+\tau^{2}A\right)\tau^{-1}\left(u_{1}-u_{0}\right)=\omega , \end{array}$$

the following formulas

(20) u0=μ,  u1=μ+τRR~ω,uk=12[Rk−1+R~k−1]μ+(R−R~)−1τ(Rk−R~k)ω−∑s=1k−1τ2iA−1/2[Rk−s−R~k−s]fs, 2≤k≤N,

were obtained in [22]. Applying formula (20) and nonlocal boundary conditions in (7), we can write formula for *μ* and *ω*

(21) μ=T−1{[[α(τ)+∑j=2Nα(tj)]τ   ×∑s=1j−1τ2iA−1/2[R~j−s−Rj−s]fs+φ]  ×[I+∑j=1Nβ(tj)τ2RR~(Rj+R~j)]  +∑j=1Nα(tj)τ2(R−R~)−1(Rj−R~j)  ×RR~[β(τ)+β(2τ)+∑j=3Nβ(tj)]τ22  ×∑s=1j−2[Rj−s−R~j−s]fs  −∑j=1Nβ(tj)τ2i(R−R~)A−1/2fj−1+ψ},

(22) ω=T−1{[I−∑j=1Nα(tj)τ2[Rj−1−R~j−1]]  ×[[β(τ)+β(2τ)+∑j=3Nβ(tj)]    ×τ22∑s=1j−2[Rj−s−R~j−s]fs    −∑j=1Nβ(tj)τ2i(R−R~)A−1/2fj−1+ψ]  +∑j=1Nβ(tj)iτ2A1/2[Rj−1−R~j−1]  ×[[α(τ)+∑j=2Nα(tj)]τ    ×∑s=1j−1τ2iA−1/2[Rj−s−R~j−s]fs−φ]}.

Hence, for the solution of nonlocal boundary value problem (7) we have formulas (20), (21), and (22).

Second, let us investigate stability of difference scheme (7). In [22], for the solution of (19) stability estimates

(23) $$\begin{array}{c} \underset{0\leq k\leq N}{\max}{||u_{k}||}_{H}\leq M\left\{\overset{N-1}{\underset{s=1}{\sum }}{||A^{-1/2}f_{s}||}_{H}\tau +{||A^{-1/2}\omega ||}_{H}+{||\mu ||}_{H}\right\}, \end{array}$$

(24) max⁡1≤k≤N||τ−1(uk−uk−1)||H+max⁡0≤k≤N||A1/2uk||H ≤M{∑s=1N−1||fs||Hτ+||ω||H+||A1/2μ||H},

(25) max⁡1≤k≤N−1||τ−2(uk+1−2uk+uk−1)||H+max⁡0≤k≤N||Auk||H ≤M{∑s=2N−1||fs−fs−1||H+||f1||H+||A1/2ω||H+||Aμ||H}

were established.

First of all, let us find estimate for ||*μ*||_*H*_. By using formula (21) and estimates (9), we obtain

(26) $$\begin{array}{c} {||\mu ||}_{H}\leq M\left\{{||\varphi ||}_{H}+\overset{N-1}{\underset{j=1}{\sum }}{||A^{-1/2}f_{j}||}_{H}\tau +{||A^{-1/2}\psi ||}_{H}\right\}. \end{array}$$

And, applying *A* ^−1/2^ to formula (22), we get

(27) A−1/2ω=T−1{[I−∑j=1Nα(tj)τ2[Rj−1−R~j−1]]    ×[[β(τ)+β(2τ)+∑j=3Nβ(tj)]     ×τ22∑s=1j−2[Rj−s−R~j−s]A−1/2fs     −∑j=1Nβ(tj)τ2i(R−R~)A−1fj−1       +A−1/2ψ]    +∑j=1Nβ(tj)iτ2[Rj−1−R~j−1]    ×[[α(τ)+∑j=2Nα(tj)]τ     ×∑s=1j−1τ2i[Rj−s−R~j−s]A−1/2fs−φ]}.

Using the triangle inequality, formula (27), and estimates (9), it follows that

(28) $$\begin{array}{c} {||A^{-1/2}\omega ||}_{H}\leq M\left\{\overset{N-1}{\underset{j=1}{\sum }}{||A^{-1/2}f_{j}||}_{H}\tau +{||A^{-1/2}\psi ||}_{H}+{||\varphi ||}_{H}\right\}. \end{array}$$

So, estimate (16) follows from estimates (23), (26), and (28). Second, applying *A* ^1/2^ to formula (21) and using estimates (9), we get estimate

(29) $$\begin{array}{c} {||A^{1/2}\mu ||}_{H}\leq M\left\{\overset{N-1}{\underset{j=1}{\sum }}{||f_{j}||}_{H}\tau +{||\psi ||}_{H}+{||A^{1/2}\varphi ||}_{H}\right\}. \end{array}$$

By using formula (22) and estimates (9), we obtain

(30) $$\begin{array}{c} {||\omega ||}_{H}\leq M\left\{\overset{N-1}{\underset{j=1}{\sum }}{||f_{j}||}_{H}\tau +{||\psi ||}_{H}+{||A^{1/2}\varphi ||}_{H}\right\}. \end{array}$$

Using estimates (24), (29), and (30), we obtain estimate (17) for the solution of (7). Third, applying *A* to formula (21) and using Abel's formula, we can write formula for *Aμ*

(31) Aμ=T−1{[[α(τ)+∑j=2Nα(tj)]τ    ×∑s=2j−112[R~j−s−Rj−s](fs−fs−1)    +(R~j−1−Rj−1)f1−(R~−R)fj−1+Aφ]    ×[I+∑j=1Nβ(tj)τ2RR~(Rj+R~j)]    +∑j=1Nα(tj)τ(Rj−R~j)    ×[τ22(RR~)2[β(τ)+β(2τ)+∑j=3Nβ(tj)]     ×∑s=2j−2[R~j−s−Rj−s](fs−fs−1)     +(R~j−2−Rj−2)f1−(R~−R)fj−2     −∑j=1Nβ(tj)fj−1+A1/2ψ]}.

It follows from formula (31) and estimates (9) that

(32) ||Aμ||H ≤M{∑s=2N−1||fs−fs−1||H+||f1||H+||A1/2ψ||H+||Aφ||H}.

Applying *A* ^1/2^ to formula (22) and using Abel's formula, we get

(33) A1/2ω=T−1{[I−∑j=1Nα(tj)τ2[Rj−1−R~j−1]]   ×[[β(τ)+β(2τ)+∑j=3Nβ(tj)]     ×τ22∑s=2j−2[R~j−s−Rj−s]     ×(fs−fs−1)+(R~j−2−Rj−2)f1−(R~−R)fj−1     +∑j=1Nβ(tj)τ2i(R−R~)fj−1+A1/2ψ]   +∑j=1Nβ(tj)iτ2[Rj−1−R~j−1]   ×[[α(τ)+∑j=2Nα(tj)]τ   ×∑s=2j−112[R~j−s−Rj−s](fs−fs−1)      +(R~j−1−Rj−1)f1−(R~−R)fj−1]−Aφ},

and using the triangle inequality and estimates (9), we obtain the estimate

(34) ||A1/2ω||H ≤M{∑s=2N−1||fs−fs−1||H+||f1||H+||A1/2ψ||H+||Aφ||H}.

Thus, estimate (18) follows from estimates (23), (32), and (34). This is the end of the proof of Theorem 3.

## 3. Stability of Second Order of Accuracy Difference Scheme

Now, we consider the second order accuracy difference scheme for approximate solution of boundary value problem (2)

(35) τ−2(uk+1−2uk+uk−1)+12Auk+14A(uk+1+uk−1)=fk,fk=f(tk), tk=kτ, 1≤k≤N−1, Nτ=1,u0=∑j=1Nα(tj−τ2)[uj+uj−12]τ+φ,(I+τ2A4)τ−1(u1−u0)−τ2(f0−Au0)  =∑j=1Nβ(tj−τ2)[uj−uj−1]+ψ.

The stability of solutions of this difference scheme is investigated under the assumption

(36) 1>∑j=1N|α(tj−τ2)|τ+∑j=1N|β(tj−τ2)|τ+∑j=1N|α(tj−τ2)|τ∑j=1N|β(tj−τ2)|τ.

**Lemma 4**

The following estimates hold [23]:

(37) $$\begin{array}{c} {||{\left(I\pm \frac{i\tau A^{1/2}}{2}\right)}^{-1}||}_{H\mapsto H}\leq 1,\\ {||R||}_{H\mapsto H}\leq 1,\;\;{||\tilde{R}||}_{H\mapsto H}\leq 1,\\ {||{\left(I\pm i\tau A^{1/2}\right)}^{-1}||}_{H\mapsto H}\leq 1,\\ {||\tau A^{1/2}{\left(I\pm i\tau A^{1/2}\right)}^{-1}||}_{H\mapsto H}\leq 1, \end{array}$$

where

(38) $$\begin{array}{c} R=\left(I-\frac{i\tau A^{1/2}}{2}\right){\left(I+\frac{i\tau A^{1/2}}{2}\right)}^{-1},\\ R=\left(I+\frac{i\tau A^{1/2}}{2}\right){\left(I-\frac{i\tau A^{1/2}}{2}\right)}^{-1}. \end{array}$$

**Lemma 5**

Suppose that assumption (36) holds. Then, the operator

(39) T=I−{α(τ2)+[α(τ2)+α(3τ2)](R+R~2)+α(3τ2)(R2+R~22)+∑j=3Nα(tj−τ2)R~j+Rj+Rj−1+R~j−12}τ2−{[β(τ2)−β(3τ2)]×(I+τ2A4)−1τ+β(3τ2)iA−1/2R2−R~22+iA−1/2∑j=3Nβ(tj−τ2)Rj−R~j−Rj−1+R~j−12}+{[β(τ2)−β(3τ2)](I+τ2A4)−1τ+β(3τ2)iA−1/2R2−R~22+iA−1/2×∑j=3Nβ(tj−τ2)Rj−R~j−Rj−1+R~j−12}×{α(τ2)+[α(τ2)+α(3τ2)]×(R+R~2)+α(3τ2)(R2+R~22)+∑j=3Nα(tj−τ2)×R~j+Rj+Rj−1+R~j−12}τ2−{[β(τ2)−β(3τ2)](R+R~2)+β(τ2)+β(3τ2)R2+R~22+∑j=3Nβ(tj−τ2)Rj+R~j−R~j−1−Rj−12}×{[α(τ2)+α(3τ2)]τ+α(3τ2)A−1/2R2−R~22i+A−1/2∑j=3Nα(tj−τ2)Rj−R~j+Rj−1−R~j−12i}τ2

has an inverse *T* ^−1^ and the following estimate is satisfied:

(40) ||T−1||H→H≤[1−∑j=1N|α(tj−τ2)|τ∑j=1N|β(tj−τ2)|τ  −∑j=1N|β(tj−τ2)|τ−∑j=1N|α(tj−τ2)|τ]−1.

**Proof**

Using formula (39), the triangle inequality, and estimates (37), we obtain

(41) ||T||H→H≥1−{|α(τ2)|+[|α(τ2)|+|α(3τ2)|]||R+R~2||H→H   +|α(3τ2)|×||R2+R~22||H→H   +∑j=3N|α(tj−τ2)|||R~j+Rj+Rj−1+R~j−12||H→H}τ2 −{[|β(τ2)|−|β(3τ2)|]||(I+τ2A4)−1||H→Hτ   +|β(3τ2)|×||A−1/2R2−R~22||H→H   +∑j=3N|β(tj−τ2)|     ×||A−1/2Rj−R~j−Rj−1+R~j−12||H→H} +{[|β(τ2)|−|β(3τ2)|]||(I+τ2A4)−1||H→Hτ   +|β(3τ2)|×||A−1/2R2−R~22||H→H   +∑j=3N|β(tj−τ2)|      ×||A−1/2Rj−R~j−Rj−1+R~j−12||H→H} ×{|α(τ2)|+[|α(τ2)|+|α(3τ2)|]||R+R~2||H→H   +|α(3τ2)|×||R2+R~22||H→H   +∑j=3N|α(tj−τ2)|||R~j+Rj+Rj−1+R~j−12||H→H}τ2 −{[|β(τ2)|−|β(3τ2)|]||R+R~2||H→H   +|β(τ2)|+|β(3τ2)|×||R2+R~22||H→H   +∑j=3N|β(tj−τ2)|||Rj+R~j−R~j−1−Rj−12||H→H} ×{[|α(τ2)|+|α(3τ2)|]τ   +|α(3τ2)|||A−1/2R2−R~22||H→H   +∑j=3N|α(tj−τ2)|   ×||A−1/2Rj−R~j+Rj−1−R~j−12||H→H}τ2≥1−{|α(τ2)|+|α(3τ2)|+∑j=3N|α(tj−τ2)|}τ −{|β(τ2)|+|β(3τ2)|+∑j=3N|β(tj−τ2)|}τ −{|β(τ2)|+|β(3τ2)|+∑j=3N|β(tj−τ2)|}τ ×{|α(τ2)|+|α(3τ2)|+∑j=3N|α(tj−τ2)|}τ≥1−∑j=1N|α(tj−τ2)|τ−∑j=1N|β(tj−τ2)|τ −∑j=1N|α(tj−τ2)|τ∑j=1N|β(tj−τ2)|τ.

Estimate (40) follows from this estimate.  Lemma 5 is proved.

**Theorem**

Let *φ* ∈ *D*(*A*), *ψ* ∈ *D*(*A* ^1/2^), and assumption (36) hold. For the solution of difference scheme (35) the following stability estimates

(42) $$\begin{array}{c} \underset{0\leq k\leq N}{\max}{||u_{k}||}_{H}\leq M\left\{\overset{N-1}{\underset{s=0}{\sum }}{||A^{-1/2}f_{s}||}_{H}\tau +{||A^{-1/2}\psi ||}_{H}+{||\varphi ||}_{H}\right\}, \end{array}$$

(43) max⁡1≤k≤N||τ−1(uk−uk−1)||H+max⁡0≤k≤N||A1/2uk||H ≤M{∑s=0N−1||fs||Hτ+||A1/2φ||H+||ψ||H},

(44) max⁡1≤k≤N−1||τ−2(uk+1−2uk+uk−1)||H+max⁡1≤k≤N||Auk+uk−12||H ≤M{∑s=1N−1||fs−fs−1||H+||f0||H+||A1/2ψ||H+||Aφ||H}

are valid, where *M* is independent of *f*_*s*_, 0 ≤ *s* ≤ *N* − 1, and *φ*,  *ψ*.

**Proof**

We obtain formula for the solution of difference scheme (35). For the solution of difference scheme

(45) τ−2(uk+1−2uk+uk−1)+12Auk+14A(uk+1+uk−1)=fk,fk=f(tk), tk=kτ,1≤k≤N−1, Nτ=1,u0=μ,  (I+τ2A4)τ−1(u1−u0)+τ2(Au0−f0)=ω,

the following formulas

(46) u0=μ,u1=(I+τ2A4)−1[(I−τ2A4)μ+τω+τ2f0],uk=12[Rk+R~k]μ+12iA−1/2[Rk−R~k](ω+τ2f0)−∑s=1k−1τ2iA−1/2[Rk−s−R~k−s]fs, 2≤k≤N,

were obtained in [22]. Applying formula (46) and nonlocal boundary conditions in (35), we obtain formulas

(47) μ=T−1{[I−[[β(τ2)−β(3τ2)](I+τ2A4)−1τ      +β(3τ2)iA−1/2R2−R~22      +∑j=3Nβ(tj−τ2)iA−1/2        ×Rj−R~j+R~j−1−Rj−12]]   ×{{α(τ2)+α(3τ2)+α(3τ2)A−1/2R2−R~22i     +∑j=3Nα(tj−τ2)A−1/2       ×Rj−R~j+Rj−1−R~j−12i}τ24f0    −α(3τ2)τ24iA−1/2(R−R~)f1−∑j=3Nα(tj−τ2)τ2    ×[∑s=1j−2τ2iA−1/2[Rj−s−R~j−s+Rj−s−1−R~j−s−1]fs     +τ2i(R−R~)fj−1]+φ}  +{[α(τ2)+α(3τ2)]τ+α(3τ2)A−1/2R2−R~22i    +A−1/2∑j=3Nα(tj−τ2)Rj−R~j+Rj−1−R~j−12i}τ2  ×{{[β(τ2)−β(3τ2)]τ22(I+τ2A4)−1     +τ2iA−1/2β(3τ2)R2−R~22     +∑j=3Nτ2β(tj−τ2)iA−1/2       ×Rj−R~j+R~j−1−Rj−12}f0     +iτA−1/2R−R~2     ×[β(3τ2)f1+∑j=3Nβ(tj−τ2)fj−1]     +iτ2A−1/2∑j=3Nβ(tj−τ2)     ×∑s=1j−2[Rj−s−R~j−s+R~j−s−1−Rj−s−1]fs+ψ}},

(48) ω=T−1{{I−[α(τ2)+[α(τ2)+α(3τ2)](R+R~2)    +α(3τ2)(R2+R~22)    +∑j=3Nα(tj−τ2)R~j+Rj+Rj−1+R~j−12]τ2}   ×{[β(τ2)−β(3τ2)]τ22(I+τ2A4)−1      +τ2iA−1/2β(3τ2)×R2−R~22      +∑j=3Nτ2β(tj−τ2)iA−1/2        ×Rj−R~j+R~j−1−Rj−12}f0   +{iτA−1/2R−R~2     ×[β(3τ2)f1+∑j=3Nβ(tj−τ2)fj−1]     +iτ2A−1/2∑j=3Nβ(tj−τ2)     ×∑s=1j−2[Rj−s−R~j−s+R~j−s−1        −Rj−s−1]fs+ψ}   +{[β(τ2)−β(3τ2)](I+τ2A4)−1     ×(I−τ2A4)−β(τ2)     +β(3τ2)R2+R~22     +∑j=3Nβ(tj−τ2)       ×Rj−Rj−1+R~j−R~j−12}   ×{{α(τ2)+α(3τ2)      +α(3τ2)A−1/2R2−R~22i      +∑j=3Nα(tj−τ2)A−1/2        ×Rj−R~j+Rj−1−R~j−12i}τ24f0      −α(3τ2)τ24iA−1/2(R−R~)f1      −∑j=3Nα(tj−τ2)τ2     ×[∑s=1j−2τ2iA−1/2       ×[Rj−s−R~j−s+Rj−s−1−R~j−s−1]fs        +τ2i(R−R~)fj−1]+φ}}.

Hence, for the solution of nonlocal boundary value problem (35) we have formulas (46), (47), and (48).

Now, let us investigate the stability of difference scheme (35). In [22], for the solution of (45) the following stability estimates

(49) $$\begin{array}{c} \underset{0\leq k\leq N}{\max}{||u_{k}||}_{H}\leq M\left\{\overset{N-1}{\underset{s=0}{\sum }}{||A^{-1/2}f_{s}||}_{H}\tau +{||A^{-1/2}\omega ||}_{H}+{||\mu ||}_{H}\right\}, \end{array}$$

(50) max⁡1≤k≤N||τ−1(uk−uk−1)||H+max⁡0≤k≤N||A1/2uk||H  ≤M{∑s=0N−1||fs||Hτ+||ω||H+||A1/2μ||H},

(51) max⁡1≤k≤N−1||τ−2(uk+1−2uk+uk−1)||H+max⁡1≤k≤N||Auk+uk−12||H ≤M{∑s=1N−1||fs−fs−1||H+||f0||H+||A1/2ω||H+||Aμ||H}

were established. Now, from formula (47) and estimates (37) it follows that

(52) $$\begin{array}{c} {||\mu ||}_{H}\leq M\left\{\overset{N-1}{\underset{s=0}{\sum }}{||A^{-1/2}f_{s}||}_{H}\tau +{||A^{-1/2}\psi ||}_{H}+{||\varphi ||}_{H}\right\}. \end{array}$$

Applying *A* ^−1/2^ to formula (48), we get

(53) A−1/2ω=T−1{{I−[α(τ2)+[α(τ2)+α(3τ2)](R+R~2)      +α(3τ2)(R2+R~22)      +∑j=3Nα(tj−τ2)         ×R~j+Rj+Rj−1+R~j−12]τ2}   ×{{[β(τ2)−β(3τ2)]τ22(I+τ2A4)−1      +τ2iA−1/2β(3τ2)R2−R~22      +∑j=3Nτ2β(tj−τ2)iA−1/2        ×Rj−R~j+R~j−1−Rj−12}A−1/2f0    +iτA−1/2R−R~2    ×[β(3τ2)A−1/2f1+∑j=3Nβ(tj−τ2)A−1/2fj−1]    +iτ2∑j=3Nβ(tj−τ2)    ×∑s=1j−2[Rj−s−R~j−s+R~j−s−1−Rj−s−1]A−1/2fs    +A−1/2ψ}    +{[β(τ2)−β(3τ2)]A−1/2R+R~2−β(τ2)      +β(3τ2)A−1/2R2+R~22      +∑j=3Nβ(tj−τ2)A−1/2        ×Rj−Rj−1+R~j−R~j−12}    ×{{α(τ2)+α(3τ2)      +α(3τ2)A−1/2R2−R~22i      +∑j=3Nα(tj−τ2)A−1/2        ×Rj−R~j+Rj−1−R~j−12i}τ24f0      −α(3τ2)τ24iA−1/2(R−R~)f1      −∑j=3Nα(tj−τ2)τ24i(R−R~)fj−1      −∑j=3Nα(tj−τ2)τ2      ×∑s=1j−2τ2iA−1/2[Rj−s−R~j−s+Rj−s−1            −R~j−s−1]fs+φ}}

and using estimates (37), we obtain

(54) $$\begin{array}{c} {||A^{-1/2}\omega ||}_{H}\leq M\left\{\overset{N-1}{\underset{s=0}{\sum }}{||A^{-1/2}f_{s}||}_{H}\tau +{||A^{-1/2}\psi ||}_{H}+{||\varphi ||}_{H}\right\}. \end{array}$$

So, using estimates (49), (52), and (54), we obtain (42) for the solution of (35). Applying *A* ^1/2^ to formula (47), we get

(55) A1/2μ=T−1{[I−[[β(τ2)−β(3τ2)](I+τ2A4)−1τ      +β(3τ2)iA−1/2R2−R~22      +∑j=3Nβ(tj−τ2)iA−1/2        ×Rj−R~j+R~j−1−Rj−12]]   ×{[α(τ2)+α(3τ2)+α(3τ2)R2−R~22i      +∑j=3Nα(tj−τ2)        ×Rj−R~j+Rj−1−R~j−12i]τ24f0     −α(3τ2)τ24i(R−R~)f1     −∑j=3Nα(tj−τ2)τ24i(R−R~)fj−1+A1/2φ     −∑j=3Nα(tj−τ2)τ2     ×∑s=1j−2τ2i[Rj−s−R~j−s+Rj−s−1         −R~j−s−1]fs}   +[[α(τ2)+α(3τ2)]τ+α(3τ2)R2−R~22i     +∑j=3Nα(tj−τ2)Rj−R~j+Rj−1−R~j−12i]τ2   ×{{[β(τ2)−β(3τ2)]τ22(I+τ2A4)−1      +τ2iA−1/2β(3τ2)R2−R~22      +∑j=3Nτ2β(tj−τ2)iA−1/2        ×Rj−R~j+R~j−1−Rj−12}f0     +iτA−1/2R−R~2     ×[β(3τ2)f1+∑j=3Nβ(tj−τ2)fj−1]     +iτ2A−1/2∑j=3Nβ(tj−τ2)     ×∑s=1j−2[Rj−s−R~j−s+R~j−s−1        −Rj−s−1]fs+ψ}}.

From the last formula and estimates (37) it follows that

(56) $$\begin{array}{c} {||A^{1/2}\mu ||}_{H}\leq M\left\{\overset{N-1}{\underset{s=0}{\sum }}{||f_{s}||}_{H}\tau +{||\psi ||}_{H}+{||A^{1/2}\varphi ||}_{H}\right\}. \end{array}$$

Using formula (48), the triangle inequality, and estimates (37), we obtain

(57) $$\begin{array}{c} {||\omega ||}_{H}\leq M\left\{\overset{N-1}{\underset{s=0}{\sum }}{||f_{s}||}_{H}\tau +{||\psi ||}_{H}+{||A^{1/2}\varphi ||}_{H}\right\}. \end{array}$$

Thus, estimate (43) follows from estimates (50), (56), and (57). Applying *A* to (47) and using Abel's formula, we obtain

(58) Aμ=T−1{[I−[[β(τ2)−β(3τ2)](I+τ2A4)−1τ      +β(3τ2)×iA−1/2R2−R~22      +∑j=3Nβ(tj−τ2)iA−1/2        ×Rj−R~j+R~j−1−Rj−12]]   ×[α(τ2)+α(3τ2)+α(3τ2)A1/2R2−R~22i     +∑j=3Nα(tj−τ2)A1/2       ×Rj−R~j+Rj−1−R~j−12i]τ24f0    −α(3τ2)×τ24iA1/2(R−R~)f1    −∑j=3Nα(tj−τ2)τ24iA1/2(R−R~)fj−1+Aφ    −∑j=3Nα(tj−τ2)τ4    ×[∑s=1j−2[(I−iτA1/22)Rj−s       +(I+iτA1/22)R~j−s]     ×(fs−fs−1)−[(I−iτA1/22)Rj−s            +(I+iτA1/22)R~j−s]f0     +2fj−2]    −∑j=3Nα(tj−τ2)τ4    ×[∑s=1j−2[(I−iτA1/22)Rj−s−1       +(I+iτA1/22)R~j−s−1](fs−fs−1)     −[(I−iτA1/22)Rj−s−1      +(I+iτA1/22)R~j−s−1]f0+2fj−2]    +[[α(τ2)+α(3τ2)]τ     +α(3τ2)R2−R~22i      +∑j=3Nα(tj−τ2)        ×Rj−R~j+Rj−1−R~j−12i]τ2    ×{[[β(τ2)−β(3τ2)]τ22A1/2(I+τ2A4)−1      +τ2iβ(3τ2)R2−R~22      +∑j=3Nτ2β(tj−τ2)i       ×Rj−R~j+R~j−1−Rj−12]f0    +iτR−R~2[β(3τ2)f1         +∑j=3Nβ(tj−τ2)fj−1]+A1/2ψ    −∑j=3Nβ(tj−τ2)τ4    ×[∑s=1j−2‍[(I−iτA1/22)Rj−s       +(I+iτA1/22)R~j−s](fs−fs−1)     −[(I−iτA1/22)Rj−s       +(I+iτA1/22)R~j−s]f0+2fj−2]    +∑j=3Nβ(tj−τ2)τ4    ×[∑s=1j−2‍[(I−iτA1/22)Rj−s−1        +(I+iτA1/22)R~j−s−1]       ×(fs−fs−1)       +[(I−iτA1/22)Rj−s−1         +(I+iτA1/22)R~j−s−1]f0     −2fj−2]}}.

From the last formula and estimates (37) it follows that

(59) ||Aμ||H≤M{∑s=1N−1||fs−fs−1||Hτ+||f0||H+||A1/2ψ||H+||Aφ||H}.

Applying *A* ^1/2^ to (48) and using Abel's formula, we get

(60) A1/2ω=T−1{[I−[α(τ2)+[α(τ2)+α(3τ2)]      ×(R+R~2)+α(3τ2)(R2+R~22)      +∑j=3Nα(tj−τ2)        ×R~j+Rj+Rj−1+R~j−12]]τ2   ×{[[β(τ2)−β(3τ2)]τ22A1/2(I+τ2A4)−1     +τ2iβ(3τ2)×R2−R~22     +∑j=3Nτ2β(tj−τ2)i        ×Rj−R~j+R~j−1−Rj−12]f0   +iτR−R~2[β(3τ2)f1        +∑j=3Nβ(tj−τ2)fj−1]+A1/2ψ   −∑j=3Nβ(tj−τ2)τ4   ×[∑s=1j−2[(I−iτA1/22)Rj−s‍      +(I+iτA1/22)R~j−s](fs−fs−1)   −[(I−iτA1/22)Rj−s    +(I+iτA1/22)R~j−s]f0+2fj−2]   +∑j=3Nβ(tj−τ2)τ4   ×[∑s=1j−2[(I−iτA1/22)Rj−s−1‍      +(I+iτA1/22)R~j−s−1]   ×(fs−fs−1)   +[(I−iτA1/22)Rj−s−1    +(I+iτA1/22)R~j−s−1]f0−2fj−2]}  +[[β(τ2)−β(3τ2)]R+R~2−β(τ2)   +β(3τ2)R2+R~22   +∑j=3Nβ(tj−τ2)Rj−Rj−1+R~j−R~j−12]  ×{[α(τ2)+α(3τ2)    +α(3τ2)A1/2R2−R~22i    +∑j=3Nα(tj−τ2)A1/2      ×Rj−R~j+Rj−1−R~j−12i]τ24f0    −α(3τ2)τ24iA1/2(R−R~)f1    +τ2iA1/2(R−R~)fj−1+Aφ  −∑j=3Nα(tj−τ2)τ4  ×[∑s=1j−2‍[(I−iτA1/22)Rj−s     +(I+iτA1/22)R~j−s](fs−fs−1)  −[(I−iτA1/22)Rj−s   +(I+iτA1/22)R~j−s]f0+2fj−2]  −∑j=3Nα(tj−τ2)τ4  ×[∑s=1j−2[(I−iτA1/22)Rj−s−1     +(I+iτA1/22)R~j−s−1](fs−fs−1)  −[(I−iτA1/22)Rj−s−1   +(I+iτA1/22)R~j−s−1]f0+2fj−2]}}.

Using the triangle inequality and estimates (37), we obtain

(61) ||A1/2ω||H ≤M{∑s=1N−1||fs−fs−1||Hτ+||f0||H+||A1/2ψ||H+||Aφ||H}.

As a result, estimate (44) follows from estimates (50), (59), and (61).  Theorem 6 is proved.

## 4. Application

The discretization of hyperbolic equation (2) with Neumann and integral or nonclassical and integral boundary conditions is carried out in two steps. First, let us define the grid sets

(62) Ω~h={x=xr=(h1r1,…,hmrm),  r=(r1,…,rm),  0≤rj≤Nj,  hjNj=1,  j=1,…,m},Ωh=Ω~h∩Ω,  Sh=Ω~h∩S.

We introduce the Banach space $L_{2h}=L_{2}({\tilde{\Omega }}_{h})$ of the grid functions

(63) $$\begin{array}{c} \varphi^{h}\left(x\right)=\left\{\varphi \left(h_{1}r_{1},\ldots ,h_{m}r_{m}\right)\right\} \end{array}$$

defined on ${\tilde{\Omega }}_{h}$ , equipped with the norm

(64) $$\begin{array}{c} {||\varphi^{h}||}_{L_{2}({\tilde{\Omega }}_{h})}={\left(\underset{x\in \bar{\Omega_{h}}}{\sum }{\left|\varphi^{h}(x)\right|}^{2}h_{1}\cdots h_{m}\right)}^{1/2}. \end{array}$$

To the differential operator *A* ^*x*^ generated by (2), we assign the difference operator *A*_*h*_^*x*^ by the formula

(65) $$\begin{array}{c} A_{h}^{x}u_{x}^{h}=-\overset{m}{\underset{r=1}{\sum }}{\left(a_{r}(x)u_{\overset{-}{x_{r}}}^{h}\right)}_{x_{r}r_{j}}+\sigma u^{h}\left(x\right) \end{array}$$

acting in the space of grid functions *u* ^*h*^(*x*), satisfying the condition *D* ^*h*^ *u* ^*h*^(*x*) = 0 for all *x* ∈ *S*_*h*_ or *u* ^*h*^(*x*) = 0, *x* ∈ *S*_1*h*_ and *D* ^*h*^ *u* ^*h*^(*x*) = 0, *x* ∈ *S*_2*h*_, *S*_*h*_ = *S*_1*h*_ ∪ *S*_2*h*_. *D* ^*h*^ *u* ^*h*^ is the approximation of $\left(\partial u/\partial \vec{n}\right)$ . It is known that *A*_*h*_^*x*^ is a self-adjoint positive definite operator in $L_{2}({\tilde{\Omega }}_{h})$ . With the help of *A*_*h*_^*x*^ we arrive at the nonlocal boundary value problem

(66) $$\begin{array}{c} \frac{d^{2}v^{h}\left(t,x\right)}{dt^{2}}+A_{h}^{x}v^{h}\left(t,x\right)=f^{h}\left(t,x\right),\;0<t<1,\;\;x\in {\tilde{\Omega }}_{h},\\ v^{h}\left(0,x\right)=\overset{1}{\underset{0}{\int }}\alpha \left(\rho \right)v^{h}\left(\rho ,x\right)d\rho +\varphi^{h}\left(x\right),\;x\in {\tilde{\Omega }}_{h},\\ \frac{dv^{h}\left(0,x\right)}{dt}=\overset{1}{\underset{0}{\int }}\beta \left(\rho \right)\frac{dv^{h}\left(\rho ,x\right)}{dt}d\rho +\psi^{h}\left(x\right),\;x\in {\tilde{\Omega }}_{h} \end{array}$$

for an infinite system of ordinary differential equations. Second, we replace problem (66) by the difference scheme

(67) uk+1h(x)−2ukh(x)+uk−1h(x)τ2+Ahxuk+1h(x)=fk+1h(x),fk+1h(x)=fh(tk+1,x), tk+1=(k+1)τ,1≤k≤N−1, Nτ=1,  x∈Ω~h,u0h(x)=∑m=1Nα(tm)umh(x)τ+φh(x), x∈Ω~h,(I+τ2Ahx)(u1h(x)−u0h(x))τ−1 =∑j=1Nβ(ρj)[ujh(x)−uj−1h(x)]+ψh(x), x∈Ω~h

of the first order accuracy in *t*. For the stability of first order of accuracy difference scheme, the following theorem is presented.

**Theorem**

Let *τ* and *h* be sufficiently small numbers. Then, the solutions of difference scheme (67) satisfy the following stability estimates:

(68) max⁡0≤k≤N||ukh||L2h+max⁡1≤k≤N||τ−1(ukh−uk−1h)||L2h+max⁡0≤k≤N||(ukh)x||L2h  ≤M1[max⁡1≤k≤N−1||fkh||L2h+||φx−h||L2h+||ψh||L2h],max⁡1≤k≤N−1||τ−2(uk+1h−2ukh+uk−1h)||L2h+max⁡1≤k≤N||(ux−k)x||L2h  ≤M1[||f1h||L2h+max⁡1≤k≤N−1||τ−1(fkh−fk−1h)||L2h     +||(φx−h)x||L2h+||ψx−h||L2h].

Here, *M*_1_ is independent of *τ*, *h*,*φ* ^*h*^(*x*), *ψ* ^*h*^(*x*), and *f*_*k*_^*h*^(*x*), 1 ≤ *k* < *N*.

The proof of Theorem 7 is based on the symmetry property of difference operator *A*_*h*_^*x*^ defined by formula (65) and on the following theorem on coercivity inequality of the elliptic difference problem.

**Theorem**

For the solutions of the elliptic difference problem

(69) $$\begin{array}{c} A_{h}^{x}u^{h}\left(x\right)=\omega^{h}\left(x\right),\;x\in \Omega_{h},\;\;D^{h}u^{h}\left(x\right)=0,\;x\in S_{h}\\ or\;u^{h}\left(x\right)=0,\;x\in S_{1h},\;\;D^{h}u^{h}\left(x\right)=0,\;x\in S_{2h} \end{array}$$

the following coercivity inequality holds [23]:

(70) $$\begin{array}{c} \overset{m}{\underset{r=1}{\sum }}{||u_{x_{r}{\overset{-}{x}}_{r}r_{j}}^{h}||}_{L_{2h}}\leq M{||\omega^{h}||}_{L_{2h}}. \end{array}$$

In addition, the second order of accuracy difference scheme

(71) τ−2[uk+1h(x)−2ukh(x)+uk−1h(x)] +12Ahxukh(x)+14Ahx[uk+1h(x)+uk−1h(x)]=fkh(x),              x∈Ω~h, fkh=fkh(tk,x),         tk=kτ, 1≤k≤N−1, Nτ=1,u0h(x)=∑j=1Nα(ρj−τ2)[ujh(x)+uj−1h(x)]τ2+φh(x), x∈Ω~h,(I+τ2Ahx2)τ−1[u1h(x)−u0h(x)]  −τ2[f0h(x)−Ahxu0h(x)] =∑j=1Nβ(ρj−τ2)[ujh(x)−uj−1h(x)]+ψh(x),f0h=fh(0,x),  fNh=fh(1,x), x∈Ω~h,

for approximately solving hyperbolic equation (2) with nonlocal integral and Neumann or nonclassical conditions is presented. The following theorem on the stability of (71) is obtained.

**Theorem 9**

Let *τ* and *h* be sufficiently small numbers. Then, the solution of difference scheme (71) satisfies the following stability estimates:

(72) max⁡0≤k≤N||ukh||L2h+max⁡1≤k≤N||τ−1(ukh−uk−1h)||L2h+max⁡0≤k≤N||(ukh)x||L2h  ≤M2[||f0h||L2h+max⁡1≤k≤N||(fkh−fk−1h)τ−1||L2h     +||φx−h||L2h+||ψh||L2h],max⁡1≤k≤N−1||τ−2(uk+1h−2ukh+uk−1h)||L2h +max⁡1≤k≤N||(ux−k)x+(ux−k−1)x2||L2h≤M2[||f0h||L2h+||τ−1(f1h−f0h)||L2h   +max⁡1≤k≤N−1||τ−1(fkh−fk−1h)||L2h   +||(φx−h)x||L2h+||ψx−h||L2h].

Here, *M*_2_ does not depend on *τ*,  *h*,  *φ* ^*h*^(*x*), and *f*_*k*_^*h*^, 0 ≤ *k* < *N* − 1.

The proof of Theorem 9 is based on the symmetry property of difference operator *A*_*h*_^*x*^ defined by formula (65) and on Theorem 8 on coercivity inequality of elliptic difference problem (69).

## 5. Numerical Examples

In this section, we apply finite difference schemes (67) and (71) to three examples which are one-dimensional hyperbolic equation with nonlocal and nonclassical conditions.

**Example**

The nonlocal boundary value problem

(73) ∂2u(t,x)∂t2−(1+x)∂2u(t,x)∂x2−ux(t,x)+u(t,x)  =f(t,x),f(t,x)=[(x+2)cos⁡x−sinx](et−1−t)+etcos⁡x,0<t<1, 0<x<π,u(0,x)=∫01e−su(s,x)ds+φ(x),  φ(x)=(1−3e−1)cos⁡x,ut(0,x)=∫01e−sus(s,x)ds+ψ(x),ψ(x)=−e−1cos⁡x,0≤x≤π,ux(t,0)=ux(t,π)=0, 0≤t≤1,

for one-dimensional hyperbolic equation with variable coefficients is considered. The exact solution of this problem is

(74) $$\begin{array}{c} u\left(t,x\right)=\left(e^{t}-1-t\right)\cos x. \end{array}$$

For the approximate solution of the problem (73), we apply finite difference schemes (67) and (71).

First, we obtain the first order of accuracy difference scheme

(75) unk+1−2unk+unk−1τ2−(1+xn)un+1k+1−2unk+1+un−1k+1h2  −un+1k+1−un−1k+12h+unk+1=f(tk+1,xn),f(tk+1,xn)=[(xn+2)cos⁡xn−sinxn]×(etk+1−1−tk+1)+etk+1cos⁡xn, Nτ=1,  xn=nh,1≤n≤M−1, Mh=π,un0−∑k=1Ne−kττunk=φ(xn), tk=kτ,  1≤k≤N−1,φ(xn)=(1−3e−1)cos⁡xn, 0≤n≤M,un1−un0−∑k=1Ne−kτ(unk−unk−1)=ψ(xn),ψ(xn)=−e−1cos⁡xn, 0≤n≤M−1,u0k=u1k, uMk=uM−1k, 0≤k≤N.

The system can be written in the matrix form

(76) $$\begin{array}{c} A_{n}u_{n+1}+B_{n}u_{n}+C_{n}u_{n-1}=D\varphi_{n},\;1\leq n\leq M-1,\\ u_{0}=u_{1},\;\;u_{M}=u_{M-1}. \end{array}$$

Here,

(77) An=[0000⋯0000an0⋯00000an⋯00⋮⋮⋮⋮⋱⋮⋮0000⋯an00000⋯0an0000⋯00](N+1)×(N+1),Bn=[1−τe−τ−τe−2τ⋯−τe−(N−1)τ−τe−Nτbcdn⋯000bc⋯0000b⋯00⋮⋮⋮⋱⋮⋮000⋯dn0000⋯cdn−1+e−τatap⋯an−e−Nτ](N+1)×(N+1),Cn=[0000⋯0000en0⋯00000en···00⋮⋮⋮⋮⋱⋮⋮0000⋯en00000⋯0en0000⋯00](N+1)×(N+1),an=−(1+xn)h2−12h,  b=1τ2,  c=−2τ2,dn=1τ2+2(1+xn)h2+1,  en=−(1+xn)h2+12h,at=1−e−τ+e−2τ,  ap=−e−2τ+e−3τ,an=−e−(N−1)τ+e−Nτ,fn=[fn0fn1fn2⋮fnN](N+1)×1,fnk+1=f(tk+1,xn)=[(xn+2)cos⁡xn−sinxn]   ×(etk−1−tk)+etkcos⁡xn, 1≤k≤N−1,fn0=(1−3e−1)cos⁡xn, 0≤n≤M,fnN=−e−1cos⁡xn, 0≤n≤M,

and *D* = *I*_*N*+1_ is the identity matrix.

Consider

(78) $$\begin{array}{c} U_{s}={\left[\begin{array}{c} u_{s}^{0}\\ u_{s}^{1}\\ u_{s}^{2}\\ \vdots \\ u_{s}^{N} \end{array}\right]}_{(N+1)\times 1},\;s=n-1,n,n+1. \end{array}$$

This type system was used by [24] for difference equations. For the solution of matrix equation (76), we will use modified Gauss elimination method. We seek a solution of the matrix equation by the following form:

(79) $$\begin{array}{c} \begin{array}{c} u_{n}=\alpha_{n+1}u_{n+1}+\beta_{n+1},\;n=M-1,\ldots ,2,1, \end{array} \end{array}$$

where *u*_*M*_ = (*I*−*α*_*M*_)^−1^ *β*_*M*_, *α*_*j*_  (*j* = 1,…, *M* − 1) are (*N* + 1)×(*N* + 1) square matrices and *β*_*j*_  (*j* = 1,…, *M* − 1) are (*N* + 1) × 1 column matrices. *α*_1_ is identity and *β*_1_ is zero matrices, and

(80) αn+1=−(Bn+Cnαn)−1An,βn+1=(Bn+Cnαn)−1(Dnφn−Cnβn),n=1,2,3,…,M−1.

Second, applying formulas

(81) 2u(0)−5u(h)+4u(2h)−u(3h)h2−u′′(0)=O(h2),2u(π)−5u(π−h)+4u(π−2h)−u(π−3h)h2−u′′(π)  =O(h2)

and using the second order of accuracy implicit difference scheme (71), we get second order of accuracy difference scheme

(82) unk+1−2unk+un−1k−1τ2−(1+xn) ×[un+1k−1−2unk−1+un−1k−14h2−un+1k−2unk+un−1k2h2   −un+1k+1−2unk+1+un−1k+14h2] −[un+1k−1−un−1k−18h+un+1k−un−1k4h+un+1k+1−un−1k+18h] +12unk+unk+1+unk−14=fnk,fnk=[(xn+2)cos⁡xn+sinxn](etk−1−tk)+etkcos⁡xn,Mh=π, xn=nh, 1≤n≤M−1, Nτ=1,tk=kτ, 1≤k≤N−1,un0=∑k=1Nτ2[e−kτunk+e−(k−1)τunk−1]+φ(xn),0≤n≤M,φ(xn)=(1−3e−1)cos⁡xn, 0≤n≤M,un1−un0τ−τ4(1+xn) ×[un+11−2un1+un−11h2+un+10−2un0+un−10h2] +τ2[−un+11−un−114h−un+10−un−104h] +τ4[un0+un1]−τ4fn0=∑k=1Ne−kτ+(τ/2)[unk+unk−1]+ψ(xn), 1≤n≤M−1,ψ(xn)=−e−1cos⁡xn, 0≤n≤M,u10−u00=h22[2u00−5u01+4u02−u03τ2+u00−φ00]u1N−u0N=h22[2u0N−5u0N−1+4u0N−2−u0N−3τ2+u0N−φ0N]u1k−u0k=h22[u0k+1−2u0k+u0k−1h2+u0k−φ0k],1≤k≤N−1,

for the approximate solutions of nonlocal boundary value problem (73). We have again (*N* + 1)×(*N* + 1) system of linear equations. We can write the system as a matrix equation (76). Here,

(83) En=[0000···000an2anan0···0000an2anan···000⋮⋮⋮⋮⋱⋮⋮⋮0000···an2ananwnwn00···000](N+1)×(N+1),Fn=[1−τ2−τe−τ−τe−2τ−τe−3τ···−τe−(N−1)τ−τ2e−Nτbndnbn0···000bndnbn···00⋮⋮⋮⋮⋱⋮⋮0000···bn00000···dnbnyntnλe−3τ/2λe−5τ/2···λe−(2N−3)τ/2−e−Nτ+(τ/2)](N+1)×(N+1),Gn=[0000···000cn2cncn0···0000cn2cncn···000⋮⋮⋮⋮⋱⋮⋮⋮0000···cn2cncnznzn00···000](N+1)×(N+1),an=−(1+xn)4h2−18h,  bn=1τ2+1+xn2h2+14,cn=−(1+xn)4h2+18h,  dn=−2τ2+1+xnh2+12,yn=−1τ+τ4+(1+xn)τ2h2+e−τ/2,tn=1τ+τ4+(1+xn)τ2h2+(e−τ−1)e−τ/2,wn=−τ24h−(1+xn)τ22h2,  zn=τ24h−(1+xn)τ22h2,  λ=(e−τ−1)fn=[fn0fn1⋮fnN](N+1)×1,fnk=f(tk,xn)=[(xn+2)cos⁡xn−sinxn]  ×(etk−1−tk)+etkcos⁡xn, 1≤k≤N−1,fn0=(1−3e−1)cos⁡xn, 0≤n≤M,fnN=−e−1cos⁡xn,   0≤n≤M,T=[λ1λ2λ3λ400⋯00000aba000⋯000000aba00⋯00000⋮⋮⋮⋮⋮⋮⋱⋮⋮⋮⋮⋮000000⋯0aba0000000⋯00aba000000⋯0λ4λ3λ2λ1](N+1)×(N+1),a=−h2τ2,  b=−1h+hτ2−h2,λ1=−1h−h2(2τ2+1),  λ2=52hτ2,  λ3=−2hτ2,    λ4=h2τ2,D=IN+1,  Us=[us0us1us2⋮usN](N+1)×1, s=n−1,n,n+1.

For the solution of the matrix equation (76), we used the same algorithm as in the first order of accuracy difference scheme. Here, *u*_*M*_ = (*α*_*M*−1_ *α*_*M*_ − 4*α*_*M*_ + 3*I*)^−1^(4*β*_*M*_ − *α*_*M*−1_ *β*_*M*_ − *β*_*M*−1_),

(84) $$\begin{array}{c} \alpha_{1}=T^{-1}\left(\frac{-1}{2}I\right),\;\;\beta_{1}=T^{-1}\varphi_{0}^{k},\;0\leq k\leq N. \end{array}$$

**Example 11**

The nonlocal boundary value problem

(85) ∂2u(t,x)∂t2−(1+x)∂2u(t,x)∂x2−ux(t,x)+u(t,x)  =f(t,x),f(t,x)=[(x+2)cos⁡x+sinx−1](et−1−t)+et(cos⁡x−1), 0<t<1,  0<x<π,u(0,x)=∫01e−su(s,x)ds+φ(x),  φ(x)=(1−3e−1)(cos⁡x−1),ut(0,x)=∫01e−sus(s,x)ds+ψ(x),ψ(x)=−e−1(cos⁡x−1), 0≤x≤π,u(t,0)=ux(t,π)=0, 0≤t≤1,

is considered. Here, we use the same procedure as in the first example. The exact solution of this problem is

(86) $$\begin{array}{c} u\left(t,x\right)=\left(e^{t}-1-t\right)\left(\cos x-1\right). \end{array}$$

Using the same manner, we can construct first order of accuracy difference scheme and it can be written in the matrix form

(87) $$\begin{array}{c} A_{n}u_{n+1}+B_{n}u_{n}+C_{n}u_{n-1}=Df_{n},\;1\leq n\leq M-1,\\ u_{0}=\vec{0},\;\;u_{M}=u_{M-1}. \end{array}$$

Here, matrices *A*_*n*_, *B*_*n*_, *C*_*n*_, and *D* are given in the previous example, and

(88) fn=[fn0fn1fn2⋮fnN](N+1)×1,fnk+1=f(tk+1,xn)=[(x+2)cos⁡x+sinx−1]×(et−1−t)+et(cos⁡x−1), 1≤k≤N−1,fn0=(1−3e−1)(cos⁡x−1), 0≤n≤M,fnN=−e−1(cos⁡x−1), 0≤n≤M.

For the solution of matrix equation (87), we will use modified Gauss elimination method. We seek a solution of the matrix equation by the following form:

(89) $$\begin{array}{c} \begin{array}{c} u_{n}=\alpha_{n+1}u_{n+1}+\beta_{n+1},\;n=M-1,\ldots ,2,1, \end{array} \end{array}$$

where *u*_*M*_ = (*I*−*α*_*M*_)^−1^ *β*_*M*_, *α*_*j*_  (*j* = 1,…, *M* − 1) are (*N* + 1)×(*N* + 1) square matrices and *β*_*j*_(*j* = 1,…, *M* − 1) are (*N* + 1) × 1 column matrices. *α*_1_ and *β*_1_ are zero matrices, and

(90) αn+1=−(Bn+Cnαn)−1An,βn+1=(Bn+Cnαn)−1(Dnφn−Cnβn),n=1,2,3,…M−1.

By using the second order of accuracy implicit difference scheme (71), we can write the matrix form

(91) $$\begin{array}{c} E_{n}u_{n+1}+F_{n}u_{n}+G_{n}u_{n-1}=Df_{n},\;1\leq n\leq M-1,\\ u_{0}=\vec{0},\;\;u_{M-2}-4u_{M-1}+3u_{M}=0. \end{array}$$

Here, matrices *E*_*n*_, *F*_*n*_, *G*_*n*_, and *D* are given in the previous example, and also *f*_*n*_ is given in the first order accuracy difference scheme. For the solution of the matrix equation (91), we used the same algorithm as in the first order of accuracy difference scheme, where *u*_*M*_ = (*α*_*M*−1_ *α*_*M*_ − 4*α*_*M*_ + 3*I*)^−1^(4*β*_*M*_ − *α*_*M*−1_ *β*_*M*_ − *β*_*M*−1_), *α*_1_ and *β*_1_ are zero matrices.

**Example 12**

In this example, the nonlocal boundary value problem

(92) ∂2u(t,x)∂t2−(1+x)∂2u(t,x)∂x2−ux(t,x)+u(t,x)  =f(t,x),f(t,x)=[(x+2)cos⁡x+sinx+1](et−1−t)+et(cos⁡x+1), 0<t<1,  0<x<π,u(0,x)=∫01e−su(s,x)ds+φ(x),φ(x)=(1−3e−1)(cos⁡x+1),ut(0,x)=∫01e−sus(s,x)ds+ψ(x),ψ(x)=−e−1(cos⁡x+1),ux(t,0)=u(t,π)=0, 0≤t≤1,  0≤x≤π

for one-dimensional hyperbolic equation is considered. The exact solution of this problem is

(93) $$\begin{array}{c} u\left(t,x\right)=\left(e^{t}-1-t\right)\left(\cos x+1\right). \end{array}$$

First, we use the first order of accuracy implicit difference scheme (67) for the approximate solutions of nonlocal boundary value problem (92) and we obtain the matrix equation

(94) $$\begin{array}{c} A_{n}u_{n+1}+B_{n}u_{n}+C_{n}u_{n-1}=Df_{n},\;1\leq n\leq M-1,\\ u_{M}=\vec{0},\;\;u_{1}=u_{2}. \end{array}$$

Here, matrices *A*_*n*_, *B*_*n*_, *C*_*n*_, and *D* are the same as in the first example, and

(95) fn=[fn0fn1fn2⋮fnN](N+1)×1,fnk+1=f(tk+1,xn)=[(x+2)cos⁡x+sinx+1]   ×(et−1−t)+et(cos⁡x+1), 1≤k≤N−1,fn0=(1−3e−1)(cos⁡x+1), 0≤n≤M,fnN=−e−1(cos⁡x+1), 0≤n≤M.

For the solution of matrix equation (94), we will use modified Gauss elimination method. We seek a solution of the matrix equation by the following form:

(96) $$\begin{array}{c} \begin{array}{c} u_{n}=\alpha_{n+1}u_{n+1}+\beta_{n+1},\;n=M-1,\ldots ,2,1, \end{array} \end{array}$$

where $u_{M}=\vec{0}$ , *α*_1_ is identity and *β*_1_ is zero matrices, and

(97) αn+1=−(Bn+Cnαn)−1An,βn+1=(Bn+Cnαn)−1(Dnφn−Cnβn),n=1,2,3,…,M−1.

Second, for the approximate solutions of nonlocal boundary value problem (92), we use second order of accuracy difference scheme (71) and the formulas

(98) $$\begin{array}{c} 3u_{0}^{k}-4u_{1}^{k}+u_{2}^{k}=0,\;1\leq k\leq N-1,\\ 10u_{0}^{k}-15u_{1}^{k}+6u_{2}^{k}-u_{3}^{k}=0,\;1\leq k\leq N-1,\\ 2u_{M}^{k}-5u_{M-1}^{k}+4u_{M-2}^{k}-u_{M-3}^{k}=0,\;1\leq k\leq N-1,\\ u_{M}^{k}=0,\;1\leq k\leq N-1. \end{array}$$

The system can be written in the following matrix form:

(99) Pnun+2+Enun+1+Fnun  +Gnun−1+Rnun−2=Dfn,u2−4u1+3u0=0→,10u0k−15u1k+6u2k−u3k=0→,uM=0→,  2uMk−5uM−1k+4uM−2k−uM−3k=0→,2≤n≤M−2.

Here, *P*_*n*_ and *R*_*n*_ zero matrices and *E*_*n*_, *F*_*n*_, *G*_*n*_, and *D* are given in the first example, and also *f*_*n*_ is given in the first order accuracy difference scheme. For the solution of matrix equation (99), we will use modified Gauss elimination method. We seek a solution of the matrix equation in the following form:

(100) un=αn+1un+1+βn+1un+2+γn+1, n=M−2,…,1,uM−1=(I−45αM−1+15αM−2αM−1)−1×(45γM−1−15αM−2γM−1−15γM−2)uM=0→,

where *α*_*j*_, *β*_*j*_ are (*N* + 1)×(*N* + 1) square matrices and *γ*_*n*_  (*j* = 1,…, *M*) are (*N* + 1) × 1 column matrices defined by

(101) $$\begin{array}{c} T_{n}=F_{n}+G_{n}\alpha_{n}+R\left(\beta_{n-1}+\alpha_{n-1}\alpha_{n}\right),\\ \alpha_{n+1}=-{\left(T_{n}\right)}^{-1}\left(E_{n}+G_{n}\beta_{n}+R\alpha_{n-1}\alpha_{n}\right),\\ \beta_{n+1}={\left(T_{n}\right)}^{-1}P_{n},\\ \gamma_{n+1}={\left(T_{n}\right)}^{-1}\left(Df_{n}-G_{n}\gamma_{n}-R_{n}\left(\alpha_{n-1}\gamma_{n}+\gamma_{n-1}\right)\right),\\ \alpha_{1}=\frac{4}{3}I,\;\;\beta_{1}=-\frac{1}{3}I,\;\;\gamma_{1}=\vec{0},\\ \alpha_{2}=\frac{8}{5}I,\;\;\beta_{2}=-\frac{3}{5}I,\;\;\gamma_{2}=\vec{0}. \end{array}$$

Using formulas (100) and (101), we can compute *U*_*n*_, 1 ≤ *k* ≤ *M* − 1.

Now, let us give the results of numerical analysis. The numerical solutions are recorded for different values of *N* = *M*, and *u*(*t*_*k*_, *x*_*n*_) represents the exact solution, and *u*_*n*_^*k*^ represents the numerical solution at (*t*_*k*_, *x*_*n*_). For their comparison, the errors are computed by

(102) $$\begin{array}{c} E_{M}^{N}=\underset{1\leq k\leq N-1,1\leq n\leq M-1}{\max}\left|u\left(t_{k},x_{n}\right)-u_{n}^{k}\right|. \end{array}$$

Thus, the results given in Tables 1, 2, and 3 show that the second order of accuracy difference scheme (71) is more accurate comparing with the first order of accuracy difference scheme (67).

## 6. Conclusion

In this paper, we presented first and second order stable difference schemes for solving the second order multidimensional hyperbolic equation with nonlocal integral and Neumann or nonclassical boundary conditions. Stability of the difference schemes do not depend on any additional condition between *h* and *τ*. The numerical results given in the previous sections demonstrate the efficiency and good accuracy of these schemes. Finally we would like to mention that this technique can be applied to get the highest order stable difference schemes.

## Figures and Tables

**Table 1:** Errors for the approximate solution of problem (73).

Difference schemes	*N* = *M* = 10	*N* = *M* = 20	*N* = *M* = 40
Difference scheme (67)	0.1983	0.0449	0.0201
Difference scheme (71)	0.056	0.0014	0.0003994

**Table 2:** Errors for the approximate solution of problem (87).

Difference schemes	*N* = *M* = 10	*N* = *M* = 20	*N* = *M* = 40
Difference scheme (67)	0.1813	0.0516	0.0202
Difference scheme (71)	0.0049	0.001	0.0002381

**Table 3:** Errors for the approximate solution of problem (94).

Difference schemes	*N* = *M* = 10	*N* = *M* = 20	*N* = *M* = 40
Difference scheme (67)	0.1498	0.0713	0.0347
Difference scheme (71)	0.0482	0.019	0.0047
